# Biomarkers in Child and Adolescent Depression

**DOI:** 10.1007/s10578-021-01246-y

**Published:** 2021-09-29

**Authors:** Weronika Zwolińska, Monika Dmitrzak-Węglarz, Agnieszka Słopień

**Affiliations:** 1 Department of Child and Adolescent Psychiatry, Poznan University of Medical Sciences, Szpitalna St. 27/33, 60-572 Poznan, Poland; 2 Department of Psychiatric Genetics, Medical Biology Center, Poznan University of Medical Sciences, Rokietnicka St. 8, 60-806 Poznan, Poland

**Keywords:** Biomarkers, Depression, Child, Adolescent

## Abstract

Despite the significant prevalence of Major Depressive Disorder in the pediatric population, the pathophysiology of this condition remains unclear, and the treatment outcomes poor. Investigating tools that might aid in diagnosing and treating early-onset depression seems essential in improving the prognosis of the future disease course. Recent studies have focused on searching for biomarkers that constitute biochemical indicators of MDD susceptibility, diagnosis, or treatment outcome. In comparison to increasing evidence of possible biomarkers in adult depression, the studies investigating this subject in the youth population are lacking. This narrative review aims to summarize research on molecular and biochemical biomarkers in child and adolescent depression in order to advocate future directions in the research on this subject. More studies on depression involving the youth population seem vital to comprehend the natural course of the disease and identify features that may underlie commonly observed differences in treatment outcomes between adults and children.

## Introduction

**Fig. 1:**
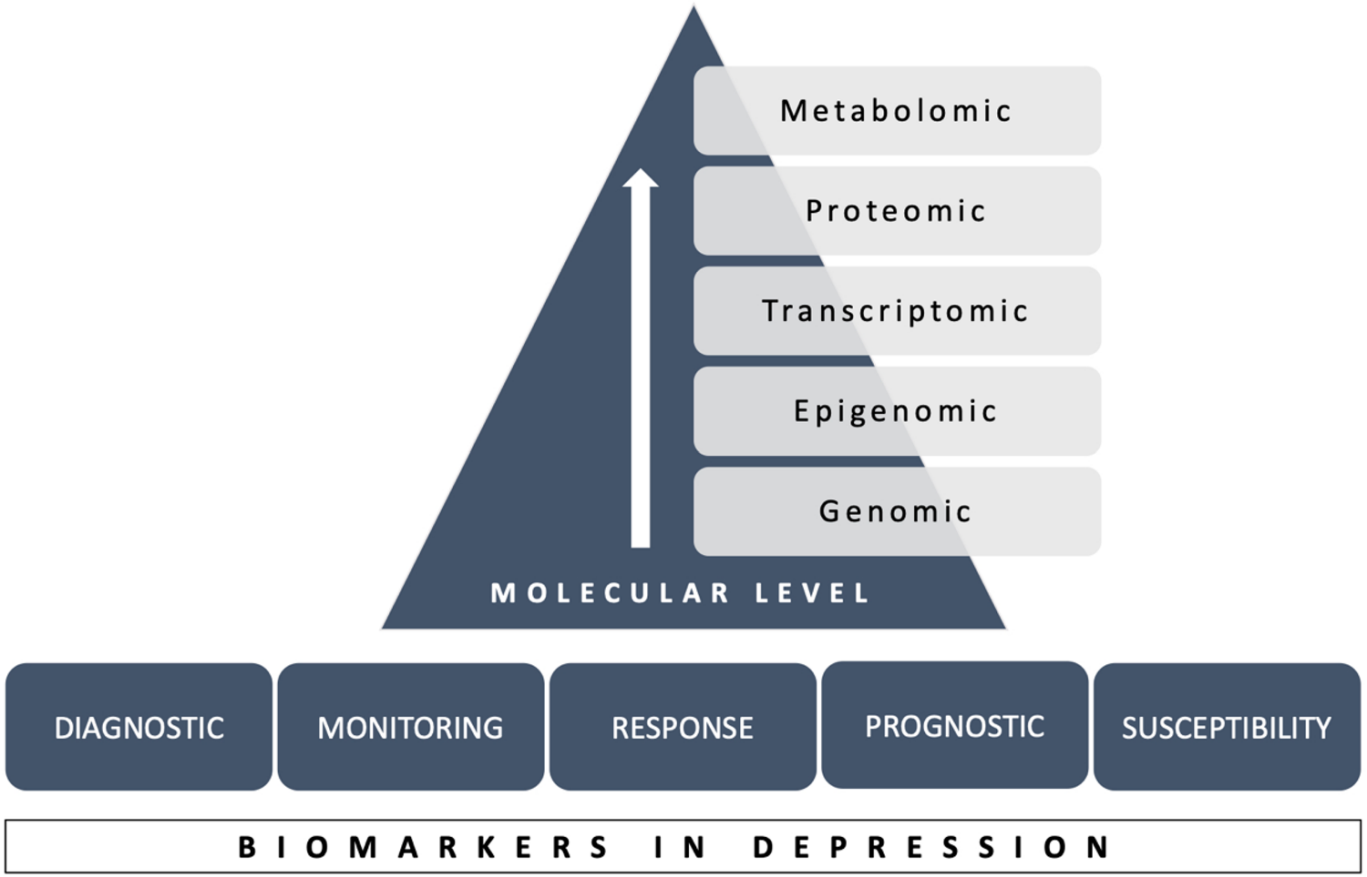
Characteristics of biomarkers in depression

Major depressive disorder (MDD) is the leading cause of disability worldwide, with the lifetime risk of developing an episode reaching 15–18%. MDD occurs throughout the lifespan, with the most probable period for the onset of the first episode extending from adolescence to middle age [1]. In its most severe form, depression may lead to suicide attempts which constitute one of the leading causes of death among adolescents in Europe [2]. Despite significant socioeconomic implications of depressive disorder, the pathophysiology of this condition remains unclear and treatment outcomes unsatisfying, with up to 60% of patients experiencing treatment resistance [3]. In light of these facts, many studies have focused on searching for biomarkers that might be utilized as specific indicators of the depression course or as prognostic factors. The FDA-NIH Biomarker Working Group [4] characterized biomarkers as “a defined characteristic that is measured as an indicator of normal biological processes, pathogenic processes or responses to an exposure or intervention”. The most promising biomarkers which have been investigated in depression derive from “-omics” technologies which involve genomics, proteomics, transcriptomics, metabolomics, and epigenetics [5] (Fig. 1).

Most studies on biomarkers in depression have been performed on the adult population. There is a significant paucity of research on this subject in the population of children and adolescents, which is striking, given the fact that approximately half of MDD diagnoses among adults derive from adolescent-onset disorder. Since early-onset episodes of depression have been associated with a worse prognosis of the future disease course, early successful treatment response is among the highest priorities for improving mental health [6]. Unfortunately, just over a half of affected adolescents respond to the initial first-choice treatment, which might be due to inadequate diagnosis or wrong treatment strategies. Diagnosing MDD is currently based on the clinical assessment of depressive symptoms, which can be tricky since the manifestation of depression among youth is usually less typical than among adults. Inventing biomarkers, which would be characteristic for children’s and adolescent’s depression, might clarify the diagnosis and improve treatment outcomes. Moreover, inventing biomarkers that could be used as predictors of antidepressant response in children would contribute to inventing more personalized and effective treatment in the youth population, which is a key to improving the prognosis of the depression course across the lifespan [6].

Unfortunately, current treatment strategies are primarily based on ‘the trial and error method,’ which prolongs time to achieve remission and therefore negatively impacts the future prognosis. A significant problem in finding an effective treatment for childhood and adolescent depression is that antidepressants were first developed for adults, and they do not necessarily have efficacy in the youth population [7]. It is worth remembering that children are not just small adults, and the knowledge on depression should not be simply extended from adult theories. Hence, the biomarkers which seem promising in diagnosing and treating adult depression might not be typical in the pediatric population, and research is needed to confirm their usefulness and characteristics in child MDD. Examining biomarkers in child and adolescent depression could broaden understanding of depression across the lifespan and eliminate potentially confounding factors that are present in adulthood, such as several recurrent episodes, comorbidities, and a history of medication.

To date, there has been no review comprehensively summarizing the current literature on molecular biomarkers in child depression. This narrative review aims to summarize potential biomarkers of depression susceptibility, diagnosis, and treatment outcome in the youth population, strictly focusing on the ones related to ‘-omics’ technologies. The work concerns most thoroughly investigated and most promising molecules. The potential biomarkers will be stratified according to the neurotransmitter, neuroendocrine, inflammatory, neurotrophic, and metabolic factors. Each biomarker will be also discussed in relation to the findings derived from adult studies.

### Biomarkers in the Neurotransmitter System

The monoamine hypothesis of depression has been the mainstay of our understanding of depression over the past 50 years. It assumes that depressed subjects display lower serotonin, dopamine, and norepinephrine levels in the neuronal circuits and that antidepressants work through increasing their bioavailability in the synaptic cleft. Research on biomarkers connected with neurotransmitters focuses mainly on the altered expression of proteins involved in the synthesis or degradation of monoamines. Serotonin transporter (5-HTT) is a protein responsible for the reuptake of serotonin from the synapse into presynaptic neurons, and it is the main target of current first-choice antidepressant drugs—SSRIs. Within the promoter of the serotonin transporter gene (*SLC6A4*), there is a serotonin-transporter-linked polymorphic region (5-HTTLPR) with a long (l) or short (s) allele resulting in respectively higher and lower *SLC6A4* gene activity [8].

According to Xia et al. [9], 5-HTTLPR is one of the most frequently investigated polymorphisms in children and adolescents with depression. The initial hypothesis that the 5-HTTLPR s allele increases the risk of depression when combined with the history of life stress was presented by Caspi et al. in [10]. This theory, however, was not confirmed in the large metaanalysis on adult patients that included 38,802 subjects overall [11]. Nevertheless, studies on the adolescent population point to the significant associations between 5-HTTLPR polymorphism, environmental stress, and risk of depression. For instance, a study performed by Hankin et al. proved that adolescents with a history of experiencing chronic peer stress were more likely to be diagnosed with a depressive episode if they carried a 5-HTTLPR s allele [12]. Similarly, Jenness et al. reported that chronic family stress predicted future increases in depressive symptoms among youth possessing the s allele of 5-HTTLPR [13]. Nobile et al. investigated the effects of a family structure and two serotonergic polymorphisms on depressive symptoms in a population of children (5-HTTLPR and tryptophan hydroxylase gene polymorphism *TPH2* G-703T) [14]. The authors concluded that both alone and in apparent gene-by-environment interaction, the 5-HTTLPR s alleles and the *TPH2* 'G variant' were associated with a higher rate of affective symptoms. The studies mentioned above suggest that s allele of 5-HTTLPR constitutes pre-existing, stable susceptibility that can potentiate the impact of environmental stress to trigger depression onset in the youth population. This association has been commonly reported among female adolescents [15–17], which is consistent with a disproportionately more significant prevalence of depression among adolescent girls than boys. Although s allele seems conducive to the development of depression in adolescence, studies show no relation between the 5-HTTLPR polymorphism and depression severity once it occurs [18].

Considering the essential part that 5-HTT plays in pharmacotherapy, it is not surprising that 5-HTTLPR polymorphism has also been widely investigated as a potential predictor of antidepressant treatment response. In their study, Kronenberg et al. aimed to examine the association between the 5-HTTLPR polymorphism and citalopram effectiveness in a group of 312 children and adolescents with MDD [19]. The authors reported that the 5-HTTLPR s/s genotype was associated with an unsatisfactory clinical outcome and suggested that this polymorphism may be a genetic marker of impaired response to citalopram in children and adolescents with depression. Additionally, Rotberg et al. suggested the cumulative effects of 5-HTTLPR polymorphism with the polymorphism in tryptophan hydroxylase gene (*TPH2*) on the clinical response to citalopram among children and adolescents with MDD [20]. They found that patients carrying the combination of *TPH2*—703G and the 5-HTTLPR l alleles were the most likely to respond to the treatment. On the other hand, the study performed by Brent et al. revealed no influence of 5-HTTLPR or *TPH1* polymorphism on antidepressant response in the group of treatment resistant depressed adolescents [21]. The association between the 5-HTTLPR polymorphism and a treatment outcome has been more widely investigated among adult patients. Although the meta-analysis performed by Taylor et al. revealed no association between 5-HTTLPR polymorphism and treatment outcomes [22], such a correlation was found in the meta-analysis performed by Porcelli et al. after stratifying the results regarding ethnicity [23]. The authors confirmed the association between the l/l genotype and a better response to SSRIs as far as the Caucasian population was concerned. Unfortunately, studies investigating the association between 5-HTTLPR polymorphism and treatment outcome in children and adolescents are scarce and more studies are needed to confirm the hypothesis proposed by Kronenberg et al. [19].

More recent studies focus on investigating the epigenetic modifications that change the expression of genes according to environmental factors in contrast to polymorphisms which remain the same throughout the lifetime. For instance, Kang et al. reported that higher SLC6A4 promoter methylation status was significantly associated with adverse life events as well as worse clinical presentation of depression, and they suggested that SLC6A4 methylation status could serve as a marker for childhood adversities and as a clinical biomarker for specific presentations of depression [24]. Interestingly, Swartz et al. found that environmental stress during adolescence was associated with an increase in methylation of the proximal promoter of the SLC6A4 gene, which predicted increases in threat-related amygdala reactivity and a later manifestation of depressive symptoms [25].

Altered expression of genes encoding proteins involved in monoamine metabolism, such as 5-HTT or TPH, might serve as a proxy of an increased risk of depression or antidepressant treatment resistance. 5-HTTLPR polymorphism is the most widely investigated polymorphism in the population of depressed youth. Despite inconclusive results derived from studies on the adult population, studies performed on the youth population suggest that 5-HTTLPR s allele might be conducive to depression, especially among female adolescents. Secondly, this allele may also be associated with worse treatment outcomes in the pediatric population. This notion may underlie commonly observed treatment resistance among depressed adolescents. However, more studies are needed to confirm the clinical usefulness of the 5-HTTLPR polymorphism in predicting the treatment outcome in the youth population. Epigenetic modifications in the promoter of the SLC6A4 gene might constitute a specific biological mechanism through which adversity contributes to an altered brain function, which, in turn, moderates the emergence of depression.

### Biomarkers in Neuroendocrine System

There is firm evidence supporting the role of hypothalamic–pituitary–adrenal (HPA) axis dysregulation in the pathophysiology of adult depression. Approximately half of the depressed adults display stress-induced hyperactivity of the HPA-axis with impaired negative feedback regulation [26]. It is reflected in persistent hypercortisolemia and non-suppression in the dexamethasone suppression test (DST). Moreover, adult studies have consistently demonstrated that a heightened cortisol response to the DST is associated with more severe depressive symptomatology, and thus, it may serve as an index of clinical severity [26]. A meta-analysis performed by Ribeiro et al. concluded that posttreatment non-suppression of cortisol on the DST could also be utilized to predict poor outcomes in depression [27]. A combined dexamethasone suppression-CRH stimulation test (DEX/CRH) has been proposed as a potentially more sensitive measure of HPA-axis dysfunction in MDD. Exaggerated cortisol response in the DEX/CRH test was found as a predictor of relapse among adult patients [28]. Moreover, the attenuation of HPA-axis measured with DEX/CRH during the first weeks of antidepressant treatment has been proposed as a predictor of treatment outcome yet with variable results [29, 30].

Given the pediatric population, it is essential to underline that HPA-axis activity differs according to the developmental period, with greater responses in adolescence and weaker activity in childhood. Physiological hyperactivity of the HPA-axis in adolescence results in higher cortisol levels and more robust HPA-axis responses than in adulthood, which is particularly important given that adolescence is when the prevalence of depression increases, especially among adolescent girls [31]. Although not every teenager develops depressive symptoms, physiologically increased activity of the HPA-axis in this period might result in increased vulnerability to mood disorders. Studies on DST responses in the youth population have yielded variable results, probably due to a large number of small-sampled studies and ununified dexamethasone doses [32]. The first systematic review of the studies on the DST in depressed youth dates back to 1989 and was performed by Casat et al. [33]. Their review involved 13 studies and included 145 children and 475 adolescents. The authors reported combined DST sensitivity for MDD in both groups to be 54.7% and the specificity to reach 78.4%. The sensitivity of the DST was higher among children than among adolescents with MDD, while the specificity of the DST appeared to be superior in the adolescent group. Remarkably, the authors concluded that children in inpatient settings were more likely to be non-suppressors than children in outpatient settings, which reflects adult findings of greater HPA-axis dysregulation associated with more severe depressive symptomatology [26]. A more recent meta-analysis conducted by Lopez—Duran et al. also supports an association between HPA-axis dysregulation and pediatric depression [34]. Based on 17 studies, the authors summarized that compared to control peers, depressed subjects tended to have a dysregulated response to DST with persistent hypercortisolemia. Regarding CRH infusion tests, most studies investigating the youth population's responses, proved no significant differences in cortisol or ACTH secretion between MDD and non-MDD groups [35–37].

Biomarkers related to HPA-axis stress response could also be found on the genomic, epigenomic, or transcriptomic levels. It has been suggested that early life stress alters the HPA-axis regulation and increases the risk of depression through epigenetic modifications in the glucocorticoid receptor gene (*NR3C1*). A prospective study performed by Humphreys et al. showed that methylation levels in the *NR3C1* gene significantly predicted the onset of MDD across adolescence and early adulthood [38]. Notably, the association remained significant after controlling for genetic variations and family history of MDD, suggesting that the methylation profile of *NR3C1* may be used as an independent, non-hereditary biomarker of MDD risk. Several studies have also shown that hypermethylation in the *NR3C1* gene was associated with the history of stress and the emergence of depressive symptoms in children and adolescents [39–41]. In line with these notions, Spindola et al. reported decreased peripheral expression of *NR3C1* in children with MDD [42].

FKBP51 is a potent inhibitor of the glucocorticoid receptor, and thus, an essential regulator of the HPA-axis stress response. Many studies have shown that minor alleles of various *FKBP5* gene’s single nucleotide polymorphisms (SNPs) increase the risk for MDD in adults, particularly rs1360780, rs9470080, rs3800373 [43, 44]. Such correlation has also been confirmed in the study performed on adolescent patients [45]. Similarly, Brent et al. found an association between rs1360780 and rs3800373 polymorphisms and the occurrence of suicidal events in the group of adolescent patients [21]. Although there is strong evidence proving that the expression of *FKBP5* modulates the response to antidepressants in adults [46, 47], no such correlation was found in the study performed on the group of adolescent patients [21]. However, the authors observed a non-significant trend for the *FKBP5* rs1360780 and rs3800373 genotypes to be associated with a lower antidepressant response rate. More studies on the youth population are needed to verify the relation between altered *FKBP5* expression and the treatment outcome in adolescent depression.

HPA-axis dysregulation might be one of the factors conducive to developing the depressive disorder in the youth population, and it can be reflected in non-suppression in the DST test. Contrary to adults, children tend to respond correctly to CRH infusion tests, suggesting that chronic hypersecretion of CRH develops with a longer disease course. Possibly, the association between HPA-axis dysregulation and depressive symptoms strengthens with age or correlates with the number of episodes. The individual response to DST or CRH infusion test could be determined by genetic factors, such as *NR3C1* and *FKBP5*. Altered expression of these genes, resulting from polymorphisms (*FKBP5*) or epigenetic modifications (*NR3C1*), seems promising as markers of susceptibility, diagnosis, or treatment outcome in child MDD; however, more studies are needed to verify their usefulness in child and adolescent depression.

### Biomarkers in Inflammation System

**Fig. 2:**
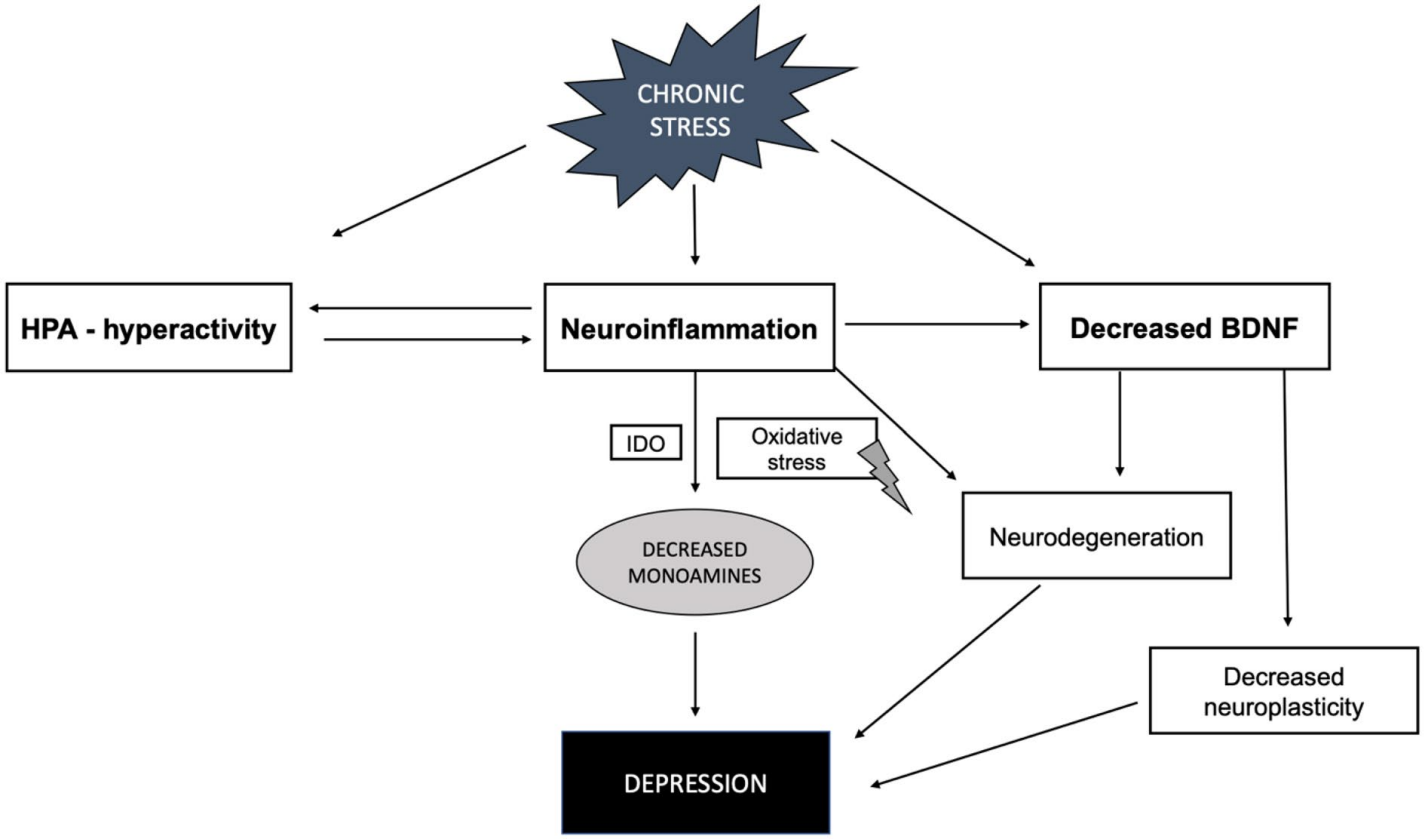
The inflammatory theory of depression

Another hypothesis explaining the pathogenesis of depression is associated with possible maladaptive inflammation processes occurring in the central nervous system due to chronic psychological or physical stress (Fig. 2). Chronic stress and preserved neuroinflammation induce bidirectional changes in the HPA-axis response system by inducing desensitization of glucocorticoid receptors and glucocorticoid resistance which, in turn, impairs the anti-inflammatory activity of glucocorticoids. Pro-inflammatory cytokines are also known to increase the activity of indoleamine 2,3-dioxygenase (IDO), which results in decreased bioavailability of tryptophan – a serotonin precursor. Moreover, it is postulated that by generating oxidative stress, the inflammatory cytokines impair the synthesis of nearly all monoamines known to be involved in the pathogenesis of depression [48]. Chronic neuroinflammation triggers excitotoxic neuronal death and impedes the production of BDNF, leading to the degeneration of neuronal circuits connected with mood regulation [49]. Studies performed on adults show that approximately one-third of patients suffering from MDD display increased blood levels of inflammatory markers. Meta-analyses of the literature point to peripheral blood levels of IL-6, TNF-α, and C-reactive protein (CRP) to be the most promising biomarkers of inflammation in adult MDD patients [50–53].

Regarding the youth population Spindola et al. [42] reported altered expression of the genes involved in the inflammatory response in children with MDD. The authors found decreased mRNA blood levels of *TNF, TNFR1*, and *IL1B* suggesting that the expression of these genes might underlie the pathophysiology of MDD in children and adolescents. Moreover, in support of the neuroinflammatory hypothesis of depression, Pandey et al. reported post-mortem expression of TNF-α, IL-1β, and IL-6 to be significantly increased in the prefrontal cortexes of teenage suicide victims [54]. Peters et al. investigated the blood levels of the same cytokines (TNF-α, IL-1β, and IL-6) in the group of treatment naïve depressed adolescents and reported significantly increased IL-6 in the studied group when compared with healthy controls [55]. In the same study, authors found TNF-α levels to be significantly increased in the subgroup of depressed patients with no history of trauma, yet no significant differences in the levels of IL-1β were reported.

Recently, two meta-analyses have addressed the association between increased peripheral cytokine levels and depressive symptoms among children and adolescents. The meta-analysis performed by D'Acunto et al. included five case–control studies and reported that compared with healthy controls, participants with depressive disorders showed a trend towards higher levels of peripheral TNF-α falling short of statistical significance [56]. However, no remarkable differences in the levels of other cytokines (i.e., IFN-gamma, IL-1b, IL-4, IL-6, IL-8, and IL-10) were detected. On the other hand, another recent meta-analysis [57], which included 22 studies and a total of 20,791 participants, revealed a positive association between depression and CRP and IL-6 levels. The authors did not confirm any association between TNF-α level and depression, although the effect size fell short of statistical significance, which was coherent with a trend observed in the previous meta-analysis [56].

Importantly, elevated levels of inflammatory markers in MDD have been associated with poorer antidepressant response, potentially predicting treatment resistance among adult patients [58]. This relation was also confirmed in the group of pediatric patients with MDD, aged 9–18, treated with fluoxetine [59]. The study revealed that pretreatment levels of TNF-α, IL-1β, and IL-6 were significantly higher in non-responders than in the group of patients who responded to treatment, which indicates that baseline levels of pro-inflammatory cytokines in child MDD might help to predict resistance to fluoxetine treatment. These findings have contributed to the idea of using anti-inflammatory agents in the treatment of depression, which might result in new treatment strategies [60]. On the other hand, a conventional antidepressant therapy was also proven to reduce the level of inflammatory markers following a successful treatment response, which suggests that in some cases, antidepressants may exert their therapeutic effect through anti-inflammatory mechanisms [61]. This trend was also observed among the adolescents in the study performed by Henje Blom et al., where the authors found that the unmedicated group of depressed female adolescents had higher IL-6 levels than the group which received antidepressants [62].

The studies mentioned above indicate a possible role of pro-inflammatory states in the pathogenesis of depression in the developmental age. The levels of IL-6 and CRP were consistently elevated in the MDD groups across different studies. The results obtained from the analysis of other pro-inflammatory cytokines such as TNF-α or IL-1β have been less conclusive and differed from the results of studies performed on adults. It might be possible that their elevated levels are not so commonly present early in life but increase further with a longer course of the disease. Notably, the baseline level of IL-6 seems promising in predicting an antidepressant treatment outcome in children and adolescent depression. More studies, however, are needed to confirm this hypothesis. Further research on this subject might contribute to inventing more personalized and more effective antidepressant treatment strategies in child and adolescent depression.

### Neurotrophic Biomarkers

Brain-derived neurotrophic factor (BDNF) is a protein from the neurotrophin family that plays an essential role in neurodevelopment by promoting the proliferation of neurons and synaptogenesis. It also stimulates neuroplastic processes in the mature brain, which involve new cell formation and elimination of unnecessary neurons [63]. The neurotrophic theory of depression assumes that environmental stress factors and mutations decrease BDNF synthesis in the brain, resulting in decreased synaptic plasticity, decreased synaptic transmission, and increased neuronal degeneration [64]. Those impairments are considered to cause specific structural changes in the areas of the brain known to be involved in cognition and mood regulation, such as the atrophy of the prefrontal cortex and hippocampal shrinkage [65]. This theory is supported by studies showing a decreased level of BDNF in the post-mortem brain samples of patients suffering from MDD [66].

One of the mechanisms considered to affect BDNF bioavailability in the brain and, therefore, underlie depression is a single nucleotide polymorphism in the *BDNF* gene (Val66Met). The *BDNF Met* allele has been associated with MDD in the population of adult and geriatric patients, while the *Val* allele has been associated with childhood-onset mood disorders [66–68]. In line with these findings, in their study, Hilt et al. concluded that in the group of adolescent females, the *Val/Val* genotype was associated with more depressive symptoms than the *Val/Met* genotype suggesting that the *Val/Val* genotype plays a role in the development of depressive symptoms in early adolescence [69]. On the other hand, Wheeler et al. proved that the *Met* allele was associated with an increased likelihood of MDD in adolescent females through effects on amygdala-cortical connectivity [70]. The study performed by Brent et al. revealed no influence of *BDNF* polymorphism on suicidal behavior or antidepressant response in the group of treatment resistant depressed adolescents [21].

In light of these discrepancies, some studies conclude that a gene–gene or gene-environment interaction is required for *BDNF* polymorphism to contribute to major depression. A large meta-analytical study based on the studies performed on adults has proven that Val66Met polymorphism of the *BDNF* gene increases the risk of depression through influencing sensitivity to exposure to stressful life events [71]. Some studies point to the same relevance of gene and environment interaction in early-life depression. For instance, a study performed by Chen et al. provided evidence of the same modulatory role of the *BDNF* Val66Met polymorphism on the relationship between stressful life events and depressive symptoms in the adolescent population [72]. Although the authors did not find any direct relationship between the *BDNF* Val66Met polymorphism and a depressive symptom score, the *BDNF Val* allele enhanced a depressive response to stress. Similarly, in the study performed by Cruz – Fuentes et al., the cumulative number of psychosocial adversities was associated with an increase in the prevalence of depression but only among the carriers of *Val/Val BDNF* polymorphism, while the possession of at least one copy of the *BDNF Met* allele was statistically linked to a resilience towards adverse life events [73]. Some studies investigated the three–way interaction model of *BDNF* Val66Met polymorphism, the serotonin transporter linked promoter region (5-HTTLPR) polymorphism, and childhood adversity in predicting the development of depression. Although some research confirmed the combined influence of these factors on early-life depression, the results were differential, and the relation was not confirmed in a representative, population-based study of adolescents [74–76].

Growing evidence suggests that epigenetic modifications are a key mechanism through which adverse life events alter the expression of stress–related genes, including *BDNF* [77]. A few studies performed on adult subjects have proven that *BDNF* gene promoter methylation profile differs in the group of patients suffering from depression compared to the healthy control [78, 79]. Some studies imply that the epigenetic profile of the *BDNF* gene also changes along with the successful antidepressant treatment, which suggests that upregulation of BDNF synthesis in the brain may be one of the mechanisms through which antidepressants exert their clinical effect [80]. Regarding the population of children, the genome-wide methylation study performed by Weder et al. demonstrated significant differences in the methylation profile in the body of the *BDNF* gene between the groups of maltreated and non-traumatized children, therefore, postulating the influence of environmental stress on the *BDNF* expression in children [81]. Regarding depressive symptoms, the authors reported that the methylation profile at six specific sites of the gene predicted the children’s depressive symptoms at uncorrected significance levels, but not after correcting for whole genome testing. However, the study did not include the methylation analysis of *BDNF* gene promoter regions which seemed most relevant in predicting depression in the studies performed on adults [78, 79].

It has been suggested that BDNF peripheral level may serve as an indicator of the BDNF concentration in the brain. Studies performed on the adult population have demonstrated that BDNF circulating level is significantly lower in blood samples of depressed patients and that effective antidepressant treatment can reverse this effect [82, 83]. These findings indicate the correlation between the BDNF blood level and depressive symptoms, making BDNF level a potential marker of depression and recovery [84]. Although mounting evidence implicates the potential of BDNF to be used as a biomarker in depression, the studies on BDNF peripheral level in the population of children and adolescents with depression are scarce and inconclusive. Some studies, however, are consistent with the findings obtained from the adult population. For instance, a study performed on 84 adolescent patients with depression revealed significantly decreased levels of BDNF compared to the healthy control (n = 64) and a significant negative correlation between BDNF level and clinical symptoms severity [85]. Notwithstanding, these findings were not confirmed in a recent study on a group of adolescent treatment naïve MDD patients (n = 95) performed by Lee et al. [86]. The authors reported no significant differences in BDNF levels between depressed and healthy subjects. Moreover, the authors reported that a decrease in BDNF level observed after two weeks of escitalopram treatment predicted a successful treatment response, which stays in opposition to the previously mentioned neurotrophic theory of depression and might suggest a different role of BDNF in the pathogenesis of depression in the developmental age. Consistently with these findings, the study on BDNF concentrations among children with ADHD symptomatology revealed no relationship between BDNF serum levels and depressive symptoms [87]. Similarly, in the study performed by Simsek et al., which aimed to investigate the BDNF levels in a group of children who developed PTSD symptoms after experiencing trauma, there were no significant differences in BDNF levels regarding depressive symptoms in the studied group [88].

On the other hand, some studies imply that alterations of BDNF expression in adolescent depression may be related to sex. For instance, Sasaki et al. reported decreased serum BDNF levels in depressed adolescent boys but not in the group of depressed adolescent girls compared to the healthy controls [89]. Furthermore, Tsuchimine et al. reported no significant differences in BDNF concentrations in the group of female treatment naïve pediatric patients diagnosed with first-episode depression [90]. Interestingly, however, Pandey et al. found decreased levels of BDNF mRNA in peripheral blood cells of pediatric depressed subjects [91]. In the same study, the authors described decreased levels of BDNF proteins in the platelets of depressed children, which was coherent with the results obtained from the group of adult depressed participants.

Although BDNF has been widely investigated in the population of depressed adults and its role as a potential biomarker of successful antidepressant treatment is nearly established, very little is known about its usefulness as a biomarker in child depression. Considering the essential role that BDNF plays in the neuroplastic and neurodevelopmental processes, one might assume that alterations in its expression may be of importance in the pathogenesis of depression occurring in the developmental age since the brain undergoes significant structural changes between childhood and adulthood. However, the studies on BDNF expression in the population of depressed children seem less coherent than in the adult population, suggesting that decreased BDNF expression is not so evident in the pediatric population. Hence, more studies are needed to verify the role of BDNF as a biomarker in depression among children and adolescents.

### Metabolomic Biomarkers

Metabolomics focuses on substrates and products of metabolism such as lipids, fatty acids, or amino acids. Studies on the adult population have revealed a connection between depressive symptoms and some specific alterations in the metabolomic profile. It has been suggested that serum cholesterol may directly influence brain lipids and the fluidity of the cell membrane, with secondary effects on serotonergic neurotransmission. Moreover, high concentrations of cholesterol upregulate a pro-inflammatory response and increase the release of IL-6 and TNFα, which is consistent with the above-mentioned inflammatory theory of depression [92]. Therefore, a hypothesis linking alterations of circulating lipid concentrations to pathophysiological pathways related to depression has been proposed. In support of this hypothesis, the meta-analyses have proven adult depression to be associated with characteristic lipid profile, mostly with increased blood levels of triglycerides (TG), very low-density lipoproteins (VLDL), and a decreased level of high-density lipoprotein (HDL) cholesterol [93, 94]. However, there have been many discrepancies between the studies. For instance, the levels of total cholesterol (TCH) and low-density lipoproteins (LDL) have been shown to be both increased and decreased in depression [94–96].

The Finnish longitudinal study performed by Elovainio et al. aimed to identify trajectories of lipid levels as predictors for depressive symptoms across childhood and early adulthood [97]. The authors investigated a sample of 824 children, who were 3, 6, or 9 years old at baseline, for the association between serum lipids (TG, TCH, LDL, HDL) and depressive symptoms during a 21-year follow-up. The results indicated that a history of steeply increasing triglycerides levels at an early age was associated with depression onset later in life. Notably, the associations were robust to adjustments for childhood BMI. However, could alterations in lipid profile be considered markers of child and adolescent depression? This question was addressed in a study performed by Katrencíková et al. [98]. The authors investigated the relationship between depressive symptoms and lipid profile in a group of depressed children and adolescents and found no significant differences in TCH, LDL, and HDL levels between the patients and a control group. However, the HDL level was inversely correlated with a CDI depressive symptoms score, which is in line with the results obtained from previous studies performed on adults [94]. The potential relationship between lipid levels and depressive symptoms could be confounded by body mass index (BMI). Increased serum lipids are often associated with obesity, which has been associated with depressive symptoms in the pediatric population [99].

**Fig. 3:**
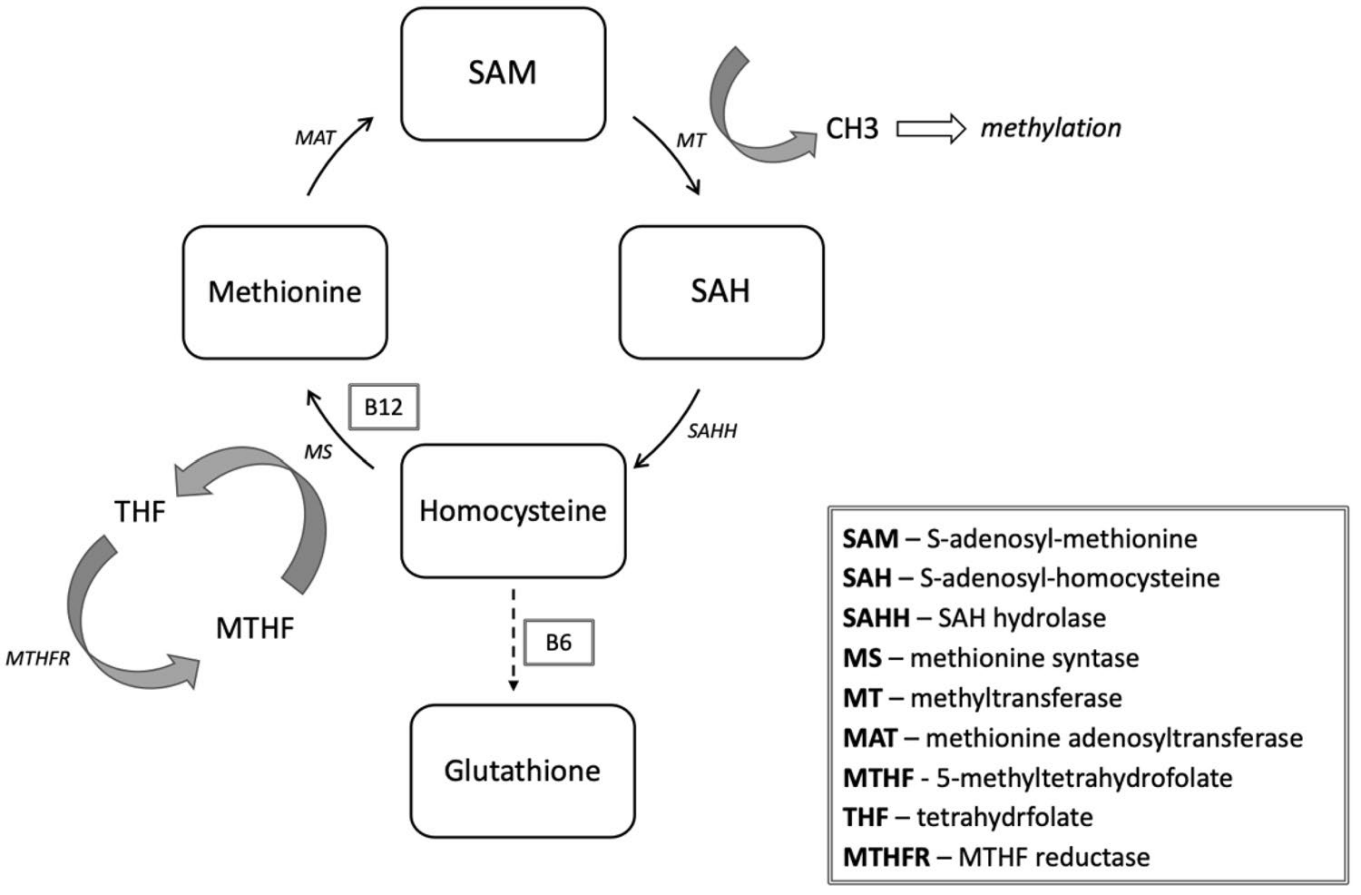
The methionine—homocysteine metabloic pathway

Another metabolomic hypothesis of depression is connected with homocysteine, a sulfurated amino acid derived from ingested methionine. The methionine-homocysteine metabolic pathway plays a fundamental role in methylation processes that are crucial in synthesizing neurotransmitters, proteins, and membrane phospholipids (Fig. 3). Reactions involved in this pathway require vitamin B12 and folate as cofactors. With a deficiency in vitamin B12 and folate levels, methylation processes are hindered, and neurotransmitter levels fall, which may underlie the development of depression [100]. Vitamin B12 and B6 are also crucial to clear homocysteine by transsulfuration into glutathione. Otherwise, the homocysteine level increases and causes a direct toxic effect to neurons and blood vessels and can induce DNA strand breakage, oxidative stress, and apoptosis [101]. Some studies support the homocysteine hypothesis of MDD as they have identified that decreased level of vitamin B12, folic acid deficiency, and hyperhomocysteinemia commonly accompany depression [102]. Moreover, it has been suggested that oral folate and B12 supplements reduce the resistance to treatment with antidepressants [103, 104]. Apart from vitamin deficiencies, hyperhomocysteinemia might also result from allelic variants of the *MTHFR* gene. Particularly, the C677T polymorphism in the *MTHFR* gene has been associated with an increased depression risk among adult patients [105, 106].

Some studies imply that alterations in homocysteine metabolic pathway may also play a role in the pathogenesis of early-onset depression. For instance, a study by Tsuchimine et al. revealed significantly decreased folic acid level in the group of children and adolescents with the first episode of depression [90]. The results obtained in a recent cross-sectional study performed on the group of 89 children and adolescents with depressive disorder are in concordance with the findings mentioned above [107]. The study revealed the vitamin B12 levels to be significantly lower and the homocysteine levels to be remarkably higher in a group of depressed children. In addition, there was a negative correlation between depression severity and vitamin B12 levels, and a positive correlation was found with homocysteine. Notably, the authors identified homocysteine levels to be higher in boys. This notion follows the results of the Taiwanese study in which authors confirmed a positive association between higher serum homocysteine levels and depressive symptoms in the population of adolescent boys [108].

The results of these studies suggest that alterations in a methionine-homocysteine metabolic pathway might be of importance in the pathophysiology of depression occurring in the developmental age, particularly in the group of adolescent boys. More studies on the youth population are needed to verify whether such metabolic disturbances may underlie the resistance towards antidepressant treatments, at least in some cases. Further investigations on this subject seem crucial to verify the practical implications of homocysteine assessment in children with depression.

Another metabolic pathway connected with mood disorders involves adenosine turnover. Adenosine impacts brain functions by influencing neurotransmission and neuromodulation mainly through presynaptic A1 and postsynaptic A2A receptors. The studies on caffeine, an A1 and A2A antagonist, provided insights into the possible effects of adenosine on mood disorders. It has been shown that moderate doses of caffeine may improve depressive symptoms, whereas excessive doses may induce ‘mania’-like symptoms [109]. Based on the potential effects of caffeine on depressive symptoms, the role of adenosine receptors in depression has been hypothesized [110]. Some studies have shown that with antidepressant treatment, the adenosine level increases, and the levels of adenosine metabolites (xanthine and hypoxanthine) decrease [111, 112]. The meta-analysis on the uric acid level in major depression, which is the end product of adenosine turnover, revealed a significantly decreased level among adult patients with MDD [113]. The results of these studies indicate that adenosine metabolism is altered in MDD and that antidepressants influence adenosine turnover.

Regarding the youth population, the results of the recent study performed by Zhou et al. suggest that perturbations in the adenosine metabolic pathway might be characteristic of children’s and adolescent’s depression [114]. The authors conducted metabolic profiling of plasma samples of 134 children and adolescents, including 52 treatment naïve MDD patients, 32 treated with antidepressants, and 50 healthy controls. Metabolites involved in purine metabolism—adenosine, inosine, and hypoxanthine—were significantly decreased in children and adolescents with MDD compared to the healthy controls, suggesting that purine degradation may be accelerated during children’s depression. Remarkably, the authors further identified inosine as a potential independent diagnostic biomarker for children’s depression, reaching an AUC of 0.999 in distinguishing the drug naïve MDD patients from healthy controls. Interestingly, the discrimination based on the inosine level was different for the drug naïve MDD group (AUC = 0.999) and the treated MDD group (AUC = 0.866), which was consistent with a previous study reporting that oral intake of inosine has antidepressant-like effects in animal models [115]. Moreover, Zhou et al. found discrepancies between adult’s and children’s metabolomic profiles in depression [114]. While inosine levels were decreased in child depression, this alteration was not characteristic of adult depression. In turn, decreased levels of tryptophan, which were found in adult MDD, were not identified in pediatric depression. This novel finding needs further investigations to confirm the specificity of inosine as a biomarker of children’s depression and its clinical implications.

### Other Biomarkers

Ciuculete et al. suggested a role of altered *MET* expression in the pathogenesis of depression among adolescents [116]. *MET* gene encodes a tyrosine kinase receptor called c-MET with a high affinity for hepatocyte growth factor (HGF). Both *MET* and *HGF* are expressed in the developing nervous system, especially in the cerebral cortex. The authors found that higher methylation levels within the *MET* gene were associated with higher depression scores and susceptibility for suicidal symptoms in the population of adolescents. Furthermore, the authors identified that methylation shifts were inversely associated with expression levels of the MET ligand HGF in blood. The authors also supported the theory of altered HGF/c-MET signaling in depression with the finding that both MET and HGF mRNA expressions were decreased in brain samples of adult depressed individuals.

In the study performed by Kaufman et al., epigenetic modifications in the Orthodenticle Homeobox 2 (*OTX2*) gene have been hypothesized to be associated with children's depression due to the role of *OTX2* in brain development [117]. *OTX2* regulates genetic networks directing the specification of dopaminergic and serotonergic neurons during embryonic development [118]. It is also engaged in neuroplastic processes in the adult brain. The hypothesis derives from the research on animals indicating that downregulation of the *OTX* gene in a reward circuit is critical in developing depressive-like behaviors in a mouse model of early-life stress [119]. In their study, Kaufman et al. reported that adversity and peripheral *OTX2* methylation profile were associated with individual differences in depression, suggesting a potential role for *OTX2* in conferring risk for MDD in children [117].

Heat-shock proteins (HSP) are multifunctional chaperone proteins that play a protective role in response to cellular stressors such as thermal or oxidative stress. Increased serum levels of anti-HSP antibodies have been suggested in the pathogenesis of psychiatric conditions such as schizophrenia [120] or bipolar disorder [121]. Bahrami et al. reported that serum antibodies against heat-shock protein 27 (anti-hsp27) were higher in the group of adolescent girls with depressive symptoms than healthy controls and that the anti-hsp27 level could predict severe depression [122]. The authors suggested the role of oxidative stress in the etiology of depression and pointed that serum anti-hsp27 antibody level may be a useful biological marker in adolescent depressive patients.

There is growing evidence supporting the role of microRNAs (miRNAs) in the pathophysiology of depression [123]. MicroRNAs are small, noncoding, single-stranded RNA transcripts that play a significant role in the post-transcriptional regulation of messenger RNA. Numerous findings suggest that circulating miRNA levels could be used as promising biomarkers of depression state or antidepressant treatment response in adult patients [124, 125]. Despite encouraging findings from the studies on adult MDD, there has been no study investigating the peripheral levels of miRNAs in pediatric depression.

### Discussion

This paper summarized the available studies on molecular biomarkers in child and adolescent depression, mainly focusing on the neurotransmitter, inflammatory, neurotrophic, neuroendocrine, and metabolomic factors. Depression is a complicated entity with heterogeneous nature and multifactorial genesis; therefore, it is expected that a single biomarker would not fully characterize it. Searching for various biochemical parameters characteristic for depression might result in introducing a panel of biomarkers that could be used to screen, diagnose, and treat the disease. Comparing to the studies performed on the adult population, the studies on biomarkers in child depression are lacking. Nevertheless, based on the studies available, we propose that certain genetic, epigenetic, proteomic, and metabolomic biomarkers might contribute to inventing the models of depression susceptibility as well as diagnosis, and treatment response in children. A summary of major findings is included in Table 1.

**Table 1:** Summary of potential molecular biomarkers in child and adolescent depression

Biomarkers	Susceptibiblity	Diagnostic	Monitoring	Prognostic*
Neurotransmitter	5-HTTLPR s/s [12–17]*SLC6A4* ↑ met [25] *TPH2* G-703T [14]	*SLC6A4* ↑ met [24]		5-HTTLPR s/s [19, 20]
Inflammatory		↓ *TNF-* α mRNA [42] *↓ IL1- β* mRNA [42] ↑ IL-6 [55, 57] ↑ CRP [57]	↓ IL-6 after treatment [62]	↑ IL-6 [59] ↑ TNF- α [59] ↑ IL-1β [59]
Neurotrophic	*BDNF* val/val [68, 69, 73]	*BDNF* val/val [69] ↓ *BDNF* mRNA [91] ↓ BDNF [85, 89, 91]	↓ BDNF after treatment [86]	
Neuroendocrine	*NR3C1* ↑ met [38–41]↓ *NR3C1* mRNA [42] *FKBP5* rs1360780, rs3800373 [21, 45]	DST non-supression [34]		
Metabolomic	↑ TG [97]	↓ HDL [98] ↓ folic acid [90, 107] ↓ B12 [107] ↑ homocysteine [107, 108] ↓ adenosine [114] ↓ inosine [114] ↓ hypoxanthine [114]		
Other	*OTX* ↑ met [117]	*MET* ↑ met [116] ↑ anti—hsp27 [122]		

↑ *met* hypermethylation, *5-HTTLPR*serotonin-transporter-linked polymorphic region, *SLC6A4* serotonin transporter gene, *TPH2* tryptophan hydroxylase gene polymorphism, *BDNF* brain-derived neurotrophic factor, *NR3C1* Nuclear Receptor Subfamily 3 Group C Member 1, *FKBP5* FK506-binding protein 51 gene, *TG* triglicerydes, *OTX* the Orthodenticle Homeobox 2 gene, *TNF-α* Tumor Necrosis Factor alpha, *IL1-β* Interleukine 1-β, *IL-6* Interleukine 6, *CRP *C-Reactive Protein, *DST* Dexamethasone Suppresion Test, *HDL* High-Density Lipoproteins, *MET* hepatocyte growth factor receptor gene, *anti-hsp27* antibodies against heat-shock protein 27

^*^Prognosis of worse treatment outcome

Studies on early-onset depression consistently point to the importance of 5-HTTLPR and BDNF Val66Met polymorphisms in the susceptibility to early-onset depression. Although 5-HTTLPR s allele [12–17] and BDNF Val allele enhanced a depressive response to stress in children across different studies [68, 69, 73], a combined three-way interaction model of *BDNF* Val66Met, 5-HTTLPR polymorphisms, and childhood adversity was not confirmed in a representative, population-based study of adolescents [74–76]. Possibly some other polymorphisms, such as *FKBP5* rs1360780, rs3800373, and *TPH2* G-703T, might also serve as predictors of increased depression risk [14, 21, 45]. The role of metabolic alterations in the pathogenesis of child depression should be considered, given the promising results of the studies summarized in Table 1.

Another attitude to explore the relation between genetics, environmental adversity and depression is through epigenetic modifications in stress-related genes. In contrast to polymorphisms which constitute a constant trait, epigenetic modifications change the expression of genes according to environmental factors. Epigenetic studies in the population of children could be beneficial in clarifying the pathogenesis of depression since the potential impact of comorbidities on epigenetic modifications is greatly reduced compared to the adult population. Available studies show that hypermethylation of the NR3C1 gene [38–41] and decreased peripheral NR3C1 mRNA expression [42] could be considered markers of increased susceptibility to early-onset depression. Other epigenetic biomarkers of increased depression risk include hypermethylation of SLC6A4 and OTX genes [25, 117]. Genetic predispositions to early-onset depression seem to be associated with different theories of depression, including neurotrophic, neuroendocrine, and neurotransmitter factors, which supports the hypothesis of their complementary rather than a contradictory role in depression. To the authors’ knowledge, there has been no study verifying the epigenetic changes following antidepressant treatment in children despite many promising results on this subject coming from adult studies [80].

Available studies show that children with depression display significant alterations in specific biochemical parameters compared to their healthy peers (Table 1). Specifically, IL-6 seems promising as a biomarker of depression state and recovery since its elevated level has been shown to decrease with successful antidepressant treatment [62]. Moreover, an increased level of IL-6 seems relevant in predicting worse treatment outcomes, which might prove useful in adjusting the optimal, individualized treatment in the future [59]. Another biomarker that could be considered a promising indicator of depression state and recovery is serum BDNF level. According to the neuroinflammatory theory of depression, IL-6 and BDNF remain closely related, as chronic neuroinflammation is considered to cause a decrease of BDNF level in the brain [49]. Indeed, some studies confirm that BDNF is significantly lower in children with depression. However, the relationship between antidepressant treatment and BDNF serum level in children remains unclear. Despite the promising role of BDNF as a marker of successful treatment in adults, the only study that addressed this question in the child population has yielded unexpected results with a decrease of BDNF level after treatment [86]. More studies on large samples are needed to verify the usefulness of serum BDNF and IL-6 as biomarkers of depression and antidepressant treatment response in children. Interestingly, inosine has been proposed as a diagnostic biomarker typical for child depression that was not found in depression among adults [114].

Moreover, when it comes to predicting the treatment outcome, studies suggest that 5-HTTLPR s allele might be associated with worse antidepressant treatment response in the pediatric population. This notion may underlie commonly observed treatment resistance among depressed adolescents [19, 20]. Interestingly, these findings do not overlap the results derived from research on adults where such correlation was not confirmed [11, 67]. However, studies on this subject in the adult population greatly outnumber those performed on the population of children.

### Summary

Despite the significant prevalence of MDD in the pediatric population, the pathophysiology of this condition remains unclear, and the treatment outcomes poor. Investigating tools that might aid in diagnosing and treating early-onset depression seems essential in improving the prognosis of the future disease course. Recent studies have focused on searching for biomarkers that constitute specific biochemical indicators of MDD susceptibility, diagnosis, or treatment outcome. Studies performed on the adult population have proposed plenty of putative biomarkers that might prove helpful in diagnosing and treating depression, whereas research on biomarkers in child and adolescent depression has been far more limited. However, the data available do not necessarily confirm the conclusions derived from studies on adults. More studies on depression involving the pediatric population seem vital to comprehend the natural course of the disease and identify features that may underlie commonly observed differences in treatment outcomes between adults and children. Identifying those features as specific biomarkers might result in more personalized and effective antidepressant treatment in the youth population.
